# Environmental and Economic Benefits of Using Pomegranate Peel Waste for Insulation Bricks

**DOI:** 10.3390/ma16155372

**Published:** 2023-07-31

**Authors:** Ayman Ragab, Nasser Zouli, Ahmed Abutaleb, Ibrahim M. Maafa, M. M. Ahmed, Ayman Yousef

**Affiliations:** 1 Department of Architecture, Faculty of Engineering, Aswan University, Aswan 81542, Egypt; ayman.ragab@aswu.edu.eg; 2 Department of Chemical Engineering, College of Engineering, Jazan University, Jazan 45142, Saudi Arabia; azabutaleb@jazanu.edu.sa (A.A.); imoaafa@jazanu.edu.sa (I.M.M.); 3 Department of Mathematics and Physics Engineering, College of Engineering at Mataria, Helwan University, Cairo 11718, Egypt; marwa_elnagar77@yahoo.com

**Keywords:** biomass waste, thermal insulation, building performance, energy efficiency

## Abstract

Rapid urbanization has negative effects on ecology, economics, and public health, primarily due to unchecked population growth. Sustainable building materials and methods are needed to mitigate these issues and reduce energy use, waste production, and environmental damage. This study highlights the potential of agricultural waste as a sustainable source of construction materials and provides valuable insights into the performance and benefits of using fired clay bricks made from pomegranate peel waste. In this study, fired clay bricks were produced using pomegranate peel waste as a sustainable building material. To optimize the firing temperature and percentage of pomegranate peel waste, a series of experiments was conducted to determine fundamental properties such as mechanical, physical, and thermal properties. Subsequently, the obtained thermal properties were utilized as input data in Design Builder software version (V.5.0.0.105) to assess the thermal and energy performance of the produced bricks. The results showed that the optimum firing temperature for the bricks was 900 °C with 10% pomegranate peel waste. The fabricated bricks reduced energy consumption by 6.97%, 8.54%, and 13.89% at firing temperatures of 700 °C, 800 °C, and 900 °C, respectively, due to their decreased thermal conductivity. CO_2_ emissions also decreased by 4.85%, 6.07%, and 12% at the same firing temperatures. The payback time for the bricks was found to be 0.65 years at a firing temperature of 900 °C. These findings demonstrate the potential of fired clay bricks made from pomegranate peel waste as a promising construction material that limits heat gain, preserves energy, reduces CO_2_ emissions, and provides a fast return on investment.

## 1. Introduction

There is a growing movement towards the use of renewable energy sources to address the world’s expanding energy demands and mounting environmental concerns [1,2]. The Kingdom of Saudi Arabia’s (KSA) strategy for growth in the year 2030 includes addressing the country’s energy crisis. Saudi Arabia’s fast economic growth over the last three decades may be attributed, in large part, to the country’s abundant natural gas and oil resources. The KSA is home to over 31 million people, and its manufacturing sector is growing at a rate of 5% to meet the country’s increasing need for energy [3]. However, the alarmingly rapid rise in national energy and oil consumption in the KSA stands out dramatically when compared to the rest of the world. Almost 70% of the projected population in 2030 will be under the age of 30, driving an annual need for new homes of 2.32 million. It is predicted that by 2040, if energy consumption drops by only 1% per year, savings of USD 35 billion will have been realized. Around 80% of the kingdom’s reserves and industrial centers are supplied with electricity from the dominant energy grid network [3,4,5].

As the Kingdom of Saudi Arabia’s population and industrial sector continue to grow, the country’s energy consumption is rapidly increasing, highlighting the need for sustainable solutions. This poses a significant challenge for the construction industry, which is responsible for a significant portion of global energy consumption and carbon emissions. To address this challenge, there is an increasing focus on using sustainable materials in the building envelope, such as bio-based materials.

Bio-based architecture, or “green architecture”, is a new approach to planning, designing, and constructing that promotes environmental sustainability by adhering to ethical, economic, and social norms at the forefront of technological development [6]. Bioarchitecture is a design approach that considers the usage of sustainable materials and renewable energy sources, such as those found in nature. Stones like lime; agricultural byproducts like potato peel, straw, and hemp; and farming byproducts like wool, etc., are all examples of naturally derived materials that are increasingly being used in contemporary architecture [7]. Paper-pulp, textile, and food-processing biowaste are only a few examples of frequently used industrial biowaste. They generate a lot of biomass waste, which is a source of interest in the quest to find innovative green materials with which to construct structures with a low impact on the environment.

Agricultural waste is a readily available residue from renewable resources and is abundant on the planet. However, the accumulation of agricultural waste in significant quantities every year poses both environmental hazards and economic challenges [8]. Therefore, the issue of waste management is a pressing concern globally, with large amounts of plant waste being dumped in landfills [9]. This waste not only takes up space but can also cause pollution of the air and water [10]. The sustainable management of agricultural waste is therefore important in terms of reducing its negative impacts on the environment and for exploring its potential for to generate economic value through the development of innovative practices and products [11]. However, there are innovative solutions to this problem, such as using plant waste as a substitute for clay in bricks [12]. This not only reduces waste but also creates a building material that is less dense and more porous, making it potentially advantageous during earthquakes. Moreover, the efficacy of using plant waste as a replacement for clay in bricks has been demonstrated in reducing the energy consumption required for climatic control in various regions [6,13,14,15]. Recent reports indicate that pomegranate production is on the rise in Saudi Arabia [16], particularly in regions such as Al-Qassim, Al-Madinah, and Al-Ahsa, which provide ideal environmental conditions for cultivation [17]. Despite the health benefits and natural color associated with pomegranate peels, they are often discarded in landfills. To address this issue, researchers have proposed using pomegranate peel waste as a substitute for clay in bricks. Pomegranate peels contain high levels of lignocellulose, a material that can enhance the properties of bricks and reduce the amount of clay required in their production [18]. By promoting the reuse of pomegranate peels and other plant waste, we can minimize waste and promote sustainable practices, thereby mitigating the harmful environmental impacts of waste disposal.

Agricultural waste is a significant environmental concern due to its potential contribution to greenhouse gas emissions and pollution if not appropriately managed. However, recent research has demonstrated that agricultural waste can be an affordable and sustainable source of raw materials for various industries, including brick manufacturing. In recent years, there has been a growing interest in the use of agricultural waste in the production of firing bricks, which are widely used in the construction industry. Various studies have examined the feasibility of using agricultural waste as a substitute for traditional raw materials in the manufacturing of firing bricks.

Huy et al. [19] conducted a study to evaluate the feasibility of using raw rice husk, bottom ash, and fly ash in the production of unburnt building bricks. This study involved the design of two mixtures with varying W/B ratios and the replacement of bottom ash content with rice husk at percentages of 0%, 3%, 6%, and 9% by mass. The results indicated that incorporating 9% rice husk led to a significant reduction in the unit weight and thermal conductivity of the bricks, with values in the range of 1.06 ÷ 1.08 T/m^3^ 0.201 ÷ 0.216 W/m.K, respectively.

Hassan et al. [20] examined the thermal characteristics of several brick samples made from clay, sludge, and sugarcane bagasse ash and assessed their energy-saving potential. The experimental findings revealed that the thermal conductivity of clay bricks was enhanced significantly by incorporating sludge and sugarcane bagasse ash as compared to traditional brick types used in Egypt. The mean thermal conductivity of the brick samples produced by mixing clay, sludge, and sugarcane bagasse ash (0.11 to 0.26 W/m. K) was found to be lower than that of traditional brick types (0.33–1.6 W/m. K). The results indicate that the predicted energy consumption for the traditional wall systems is 7525 kWh, whereas the proposed wall system, i.e., walls constructed by incorporating sludge and sugarcane bagasse ash into the clay brick, has an annual energy consumption of 6285 kWh, resulting in an overall reduction of 16.5% in energy consumption. Mehrzad et al. [15] utilized environmentally friendly sugarcane bagasse waste to create fiber samples of varying densities and thicknesses and tested them for thermal insulation and sound absorption. The thermal conductivity of the samples ranged from 0.034 to 0.042 W/mK.

Aravind et al. [14] reported the creation of a sustainable exterior wall panel with thermal insulation and its mechanical, thermal, and durability features. Foam concrete and rice husks were utilized to create the wall panel instead of fly ash, which is typically used to create cement. The created wall panels’ thermal conductivity and durability were significantly affected by the percentage of rice husk and fly ash present in the material. Marques et al. [21] developed a new polymer-based composite material that utilized waste materials from rice husk and expanded cork industries. Boards made from a variety of composite mixtures were tested for their mechanical, thermal, and acoustic qualities. The results indicate that construction solutions based on these composite materials may be employed in buildings to minimize energy consumption during the lifecycle of a building.

In a study conducted by Ghorbani et al. [10], the effect of potato peel powder (PPP) on sound insulation and other physicochemical parameters was investigated. Results showed that the addition of 7% dried PPP resulted in improved compressive strength, apparent dry density, saturated density, and thermal conductivity. These findings suggest that the use of dried PPP in building materials can have several practical implications in construction. Incorporating 7% dried PPP into bricks can potentially reduce the weight of structures, making them more cost-effective and easier to transport. Moreover, the sound insulation properties of the bricks can be beneficial in partition walls, which can improve the acoustic performance of buildings. Additionally, the study provides a cost-effective solution for crop residue management. Ramos et al. [22] compared the thermal properties and environmental effects of two corncob particleboards made using different glue binders and found that both could be used as sustainable construction materials for wall thermal insulation. Chee-Ming [23] utilized oil palm fruit and pineapple leaves to create unfired and fired clay bricks, discovering that fibers significantly reduce porosity in burned bricks compared to unfired specimens while maintaining the same strength. Elinwa [24] used sawdust ash to create lightweight bricks, recommending burning the bricks at 600 °C for good porosity and reasonable strength. J. Vėjeliene et al. [25] investigated straw insulation and found that laboratory-produced samples with most straw perpendicular to heat flow had lower thermal conductivity than plant specimens obtained from straw bales and rolls with most straw parallel to heat flow.

Huixia et al. [26] conducted a study to investigate the micro–macro characteristics of sustainable mortar containing construction waste fines (0–0.15 mm) as a replacement for cement and sand. The construction waste fines utilized in the study included both waste concrete fines (WCF) and waste brick fines (WBF). The results revealed that the incorporation of WCF as cement replacement led to a reduction in the strength and permeability resistance of the blended mortar. However, the addition of WBF content initially improved the strength and permeability resistance of the mortar, although further increases in WBF content resulted in a decline in these properties. However, this study ignores all thermal properties of the produced mortar.

Ahmed et al. [6] conducted a study evaluating the effectiveness of incorporating different percentages of pomegranate peel waste (5%, 7.5%, 10%, and 15%) into clay bricks used for the external walls of social residential buildings in New Aswan City, Egypt. The study showed a gradual increase in energy savings with an increase in the percentage of pomegranate peel waste. However, the study did not investigate the effect of varying firing temperatures on the properties of the bricks.

Saudi Arabia experiences extreme temperatures during the summer months, with daytime temperatures often exceeding 40 °C and nighttime temperatures remaining above 30 °C. As a result, air conditioning is essential for maintaining comfortable indoor temperatures, which can lead to high energy consumption. Another factor contributing to the increased energy consumption for cooling in Saudi buildings is inefficient building design. Many buildings in Saudi Arabia are constructed with inadequate insulation, which can result in heat gain during the day and heat loss at night.

The purpose of this article is to investigate the impact of replacing traditional brick with a newly proposed brick containing 10% pomegranate peel waste fired at different temperatures (700 °C, 800 °C, and 900 °C) on the indoor thermal performance and energy consumption of a residential building located in Jazan City, Saudi Arabia. The study aims to assess the potential benefits of using this new brick in terms of improving indoor thermal comfort and reducing the energy required for cooling purposes, with the goal of promoting sustainable building practices.

## 2. Materials and Methods

**Figure 1:**
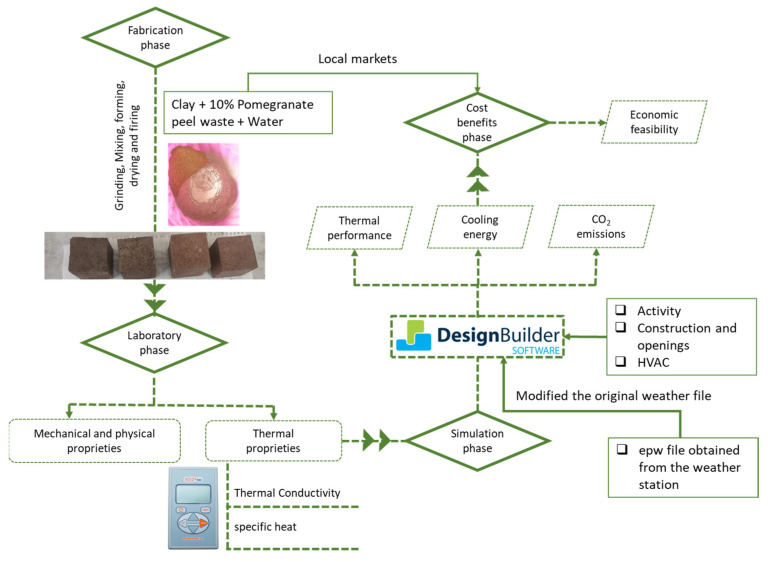
The methodological framework of the study.

The primary objective of this research was to investigate the effectiveness of integrating 10% pomegranate peel waste (PPW) into fired clay bricks for use in external walls within the Jazan region of Saudi Arabia. The research was conducted in four phases, with a graphical illustration of the research work flow presented in Figure 1.

The first phase involved the fabrication of brick samples at varying firing temperatures; the resulting samples are recorded in Table 1. In the second phase, the produced brick samples were subjected to rigorous mechanical, physical, and thermal testing. The third phase entailed the utilization of Design Builder simulation software to evaluate the potential energy savings that could be achieved using the developed brick samples. Finally, in the fourth phase, the cost-effectiveness of the proposed building materials was assessed.

### 2.1. Study Area

Jazan City, the capital of the Jazan province in southwestern Saudi Arabia, is characterized by hot and humid climatic conditions, with average high temperatures reaching 33.5 °C in June. Conversely, January is the coldest month of the year, with average temperatures falling to as low as 25.7 °C. Precipitation levels in Jazan range from 1 mm to 19 mm, with the lowest precipitation level in June and the highest levels in the wettest month of the year.

**Figure 2:**
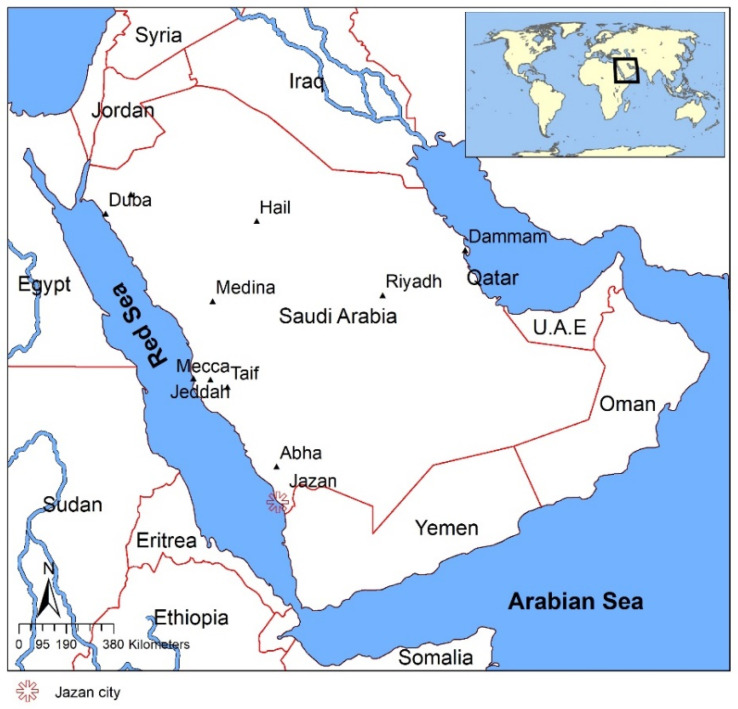
Location of Jazan City.

Figure 2 depicts the location of Jazan City in KSA. The present study aims to investigate the potential benefits of incorporating pomegranate waste in clay bricks at a concentration of 10% to reduce energy consumption and enhance thermal performance in buildings located in Jazan. By utilizing clay bricks developed from pomegranate waste, this study seeks to address the issue of uninsulated walls in Saudi Arabian buildings and contribute to the promotion of sustainable building practices in the region.

### 2.2. Raw Materials and Fabrication Process

#### 2.2.1. Materials

**Figure 3:**
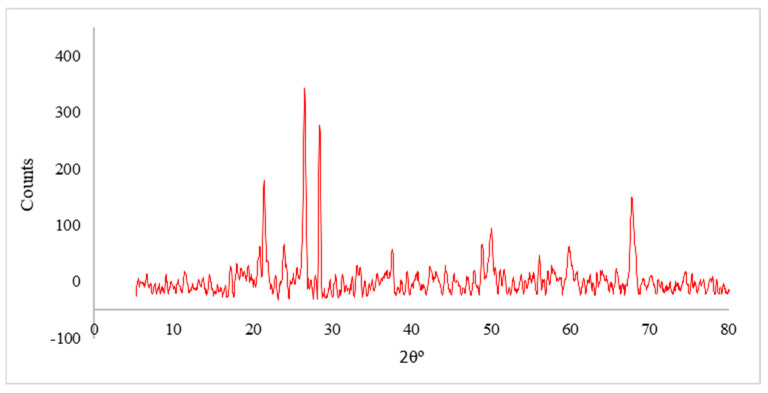
X-ray of the clay.

The main raw material used in this study for brick production was clay, obtained from the Aswan region of Upper Egypt. The studied clay exhibited similarities to the clay used in brick manufacturing in KSA. PPW waste was collected from local juice shops. After drying under normal sunlight, it was ground to achieve sufficient particle powder, then dried in an oven at 100 °C for two days to obtain fully dried powder before being mixed with clay. Table 2 explains the XRF of raw materials, which indicates high loss on ignition (LOI) of PPW, which is attributed to its high organic content. X-ray diffraction (XRD) was utilized to ascertain the mineral composition of the clay. Based on the XRD analysis, the predominant components of the clay material are silica and alumina. Figure 3 presents an analysis of the X-ray results of the utilized clay.

#### 2.2.2. Brick Preparation

The brick samples were prepared according to our previous report [27]. Typically, brick samples were prepared using fine-dried PPW powder as a partial replacement for clay in the bricks and clay. The raw materials were combined to make a homogeneous dry mixture; then, three samples from each investigation were examined to find the average value of the investigated parameter. The clay was replaced by different percentages of PPW (0–15 wt.% of PPW) [27]. The mixtures were mixed in dry conditions for 2 min to obtain a consistent dry mixture. The aforementioned mixtures were then blended again using an electric mixer with the addition of 20% water to improve the compression and consistency of the samples. The formed paste was molded in 50 × 50 × 50 mm cubic steel molds. Then, a hydraulic pressure of 10 MPa with a 0.2 mm/min press rate was applied to press the samples. Next, to ensure total moisture content elimination, brick samples were oven-dried for 6 h at 120 °C, then cooled to room temperature. Finally, the produced brick samples were fired at different temperatures (700 °C, 800 °C, and 900 °C) for four hours at 10 °C/min.

#### 2.2.3. Characterization

Characterization (compressive strength (CS), bulk density (BD), cold-water absorption (WA), apparent porosity (AP), and thermal conductivity) of the fabricated brick membranes was conducted according to an identical procedure described in our recent publication [27].

### 2.3. Thermal Properties Tests

The thermal conductivity values of the brick specimens were determined using a KD2 Pro Thermal Properties Tester, which adheres to the guidelines outlined in ASTM D 5334 [28]. The transient heat conduction method was employed to obtain a digital recording of thermal conductivity. This method involves applying a heat pulse to the specimen and measuring the resulting temperature change over time using thermocouples. The temperature change over time was then analyzed to calculate the thermal conductivity of the specimens.

It is important to note that the ambient temperature at the time of testing is a crucial factor to consider in thermal conductivity measurements because temperature has a significant impact on thermal conductivity, and measurements taken at various temperatures can yield different results. To minimize the effect of temperature on the measurements, the specimens were conditioned to a standard temperature of 23 °C prior to testing. Additionally, each specimen was tested three times to ensure the accuracy and repeatability of the results.

### 2.4. Simulation Procedures

The daily schedule of the traditional Saudi Arabian lifestyle incorporates holidays, work hours, and other events. Several festivals are celebrated throughout the year in Saudi Arabia. As mandated by Saudi Arabian law, employers are required to abide by the country’s standard work schedule, which runs from 8:00 a.m. to 3:00 p.m. from Sunday to Thursday. Recent demographic data [29] indicate that the average Saudi Arabian family comprises ten members, with building residents usually ranging from 15 to 65 years of age. The building schedule was designed with consideration of holidays and the typical weekday routine of building residents, who are expected to be at their workplaces during weekdays. Furthermore, during holidays, more people may be present in the building, which could lead to higher energy consumption for heating, cooling, and other activities. To simulate actual family routines, the simulation program was fed with relevant data; all the necessary input data for the simulation software are presented in Table 3.

#### 2.4.1. Model Description

**Figure 4:**
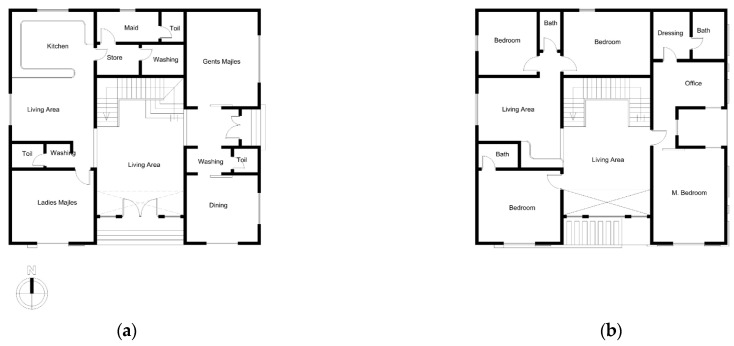
The residential building model: (**a**) first floor; (**b**) second floor.

Weather conditions such as temperature and humidity can impact the energy demands of buildings. In hot and arid climates, the energy requirements for cooling of buildings can be significant, which shows the importance of using materials with better insulation properties to reduce energy usage for both heating and cooling. As a result, this study aims to investigate the energy demand required to cool a family home located in the city of Jazan using a new insulated brick. The residential model under consideration features a two-story building with a total area of 240 m² designed to accommodate up to ten individuals comfortably. The architectural designs for this model are depicted in Figure 4. It is worth noting that this residential model can be found in various locations throughout the city of Jazan and shares similar attributes, including the use of concrete construction techniques that comply with the building thermal insulation guidelines outlined in the Saudi Building Code (SBC) [30].

#### 2.4.2. Weather Data Collection

The U.S. Department of Energy (DOE) website provides the 2002 EPW (energy plus weather) file for the Jazan climate zone in the modeling software Design Builder version (V.5.0.0.105). The EPW file is a textual CSV file containing hourly weather data for the research site throughout the year. As noted in prior research studies [31,32,33,34], we replaced the default weather data file in the software with data obtained from the weather station at Jazan University, which reflects the local climate conditions in the year 2022.

The objective was to create an environment that closely resembles the conditions observed in reality. To access the weather station’s extracted data, the original EPW file had to be converted to a CSV file, which was a crucial step for obtaining accurate information. The dry-bulb temperature, relative humidity, global radiation, wind speed, and wind direction were used as inputs, with all measurements taken at the local weather station. The dew point and direct radiation were calculated using Element software Version (1.0.6). The newly generated EPW file was then utilized to import the updated CSV file into the Design Builder simulation software for the study.

#### 2.4.3. Model Validation

To ensure the accuracy of the simulation results, a comparative analysis was carried out between the simulated data and the actual observations of air temperature. Specifically, field measurements were conducted in a selected bedroom located on the southwestern side of the investigated building on June 21; the first-floor bedroom air temperature was compared to verify the base case model.

**Figure 5:**
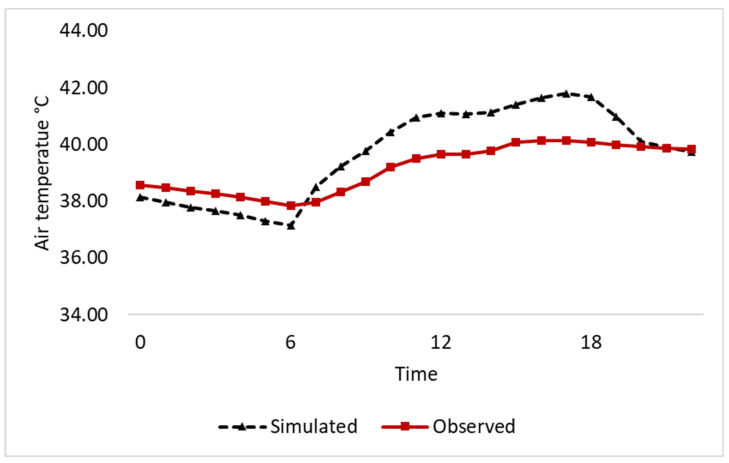
Validation of the investigated building model.

The Hobo U12 data logger was utilized to record the temperatures and relative humidity of the selected bedroom throughout the day, which included work hours from 8:00 a.m. to 5:00 p.m., Sunday through Thursday, while the remaining hours were assumed to represent typical residential occupancy. The difference between the calculated and observed air temperatures in the studied bedroom varied from 0.08% to 4.61% throughout the day, which is within the acceptable margin of less than 5% [35]. The model validation is presented in Figure 5.

### 2.5. Electricity Prices

The monthly expenses associated with energy consumption are presented in Table 4, with the cost illustrated in Saudi Arabian riyal (SAR). The tariff utilized to calculate the energy consumption expenses is determined by the Saudi Electricity Company (SEC) for the residential sector. The SEC tariff system is designed to consider various factors, including the type of customer, the amount of energy consumed, and the time of day when the energy is utilized. The tariff system is periodically reviewed and updated to ensure its accuracy and alignment with the prevailing economic conditions and market dynamics. The use of the SEC tariff system to calculate the monthly energy consumption expenses in this study provides a reliable and consistent basis for evaluating the cost-effectiveness of the proposed building materials and energy efficiency measures.

## 3. Results and Discussions

The present study is designed to investigate the efficacy of utilizing bricks made from pomegranate peel in enhancing the thermal performance and cost-effectiveness of building structures. The study is divided into four distinct parts, each addressing a specific aspect of the building’s thermal performance and energy efficiency.

The first part of the study focuses on evaluating the mechanical properties and physical characteristics of bricks. This involves assessing the compressive strength, water absorption, and density of the bricks, among other relevant parameters that can affect their performance in building applications. The second part of the study is concerned with investigating the thermal conductivity of bricks. This involves measuring the rate at which heat is transferred through the bricks and assessing their insulation properties.

The third part of the study presents simulations of the building’s interior thermal performance, cooling energy consumption, and CO_2_ emissions. This involves utilizing advanced modeling software to simulate the thermal behavior of the building under different scenarios and evaluate the potential benefits of incorporating pomegranate peel bricks in building construction. The fourth and final part of the study assesses the cost-effectiveness of utilizing bricks made from pomegranate peel to maintain appropriate indoor temperatures. This involves analyzing the cost implications of utilizing pomegranate peel bricks compared to conventional building materials and assessing the potential for long-term cost savings associated with improved thermal performance and reduced energy consumption.

### 3.1. Properties of Bricks: A Mechanical and Physical Assessment

This study focuses on the use of (PPW) as a replacement for clay in the fabrication of bricks. The study measured the effects of different percentages of PPW on the density, compressive strength, water absorption, and apparent porosity of the bricks.

Table 5 summarizes the density, compressive strength, water absorption, and apparent porosity of brick samples with different PPW percentages fired at 900 °C. As expected, the bulk density decreased when the PPW content increased in the brick samples. It is well demonstrated that the bricks fabricated with PPW have lower densities, varying between 1922 and 1348 kg/m^3^ for 0% and 15% PPW contents, respectively, which corresponds to a decrease of about 29.9% in the density. A 17.2% decrease was obtained with the addition of 10% PPW. This may be due to the fact that during the firing of PPW bricks, more material was lost due to the decomposition of carbonaceous matter [36]. Replacing PPW with clay decreases the compressive strength of bricks in most low-rise buildings, and the acceptable compressive strength is about 8.6 MPa according to ASTM C 62 [37]. Thus, it can be easily seen that all the tested bricks overwhelmingly satisfy this limit, except those replaced with 15% PPW. Nevertheless, 10% PPW is the highest amount that can be added to achieve the specifications and may be the best choice among the single-component replacement groups.

Different amounts of PPW addition and firing at different temperatures impact the compressive strength of the produced bricks. This can be explained by the size of the capillary channels and voids that are created by porosity in the brick. Additionally, the amount of clay inside the brick sample decreases, which is a key reason why the compressive strength decreases as the PPW proportion rises and the firing temperature decreases. The samples with a compressive strength between 2.5 and 3.8 MPa, referred to as Earth Blocks Class 2 (EB2), can be used in low-height construction, where loads are lower. They can also be employed in secondary buildings in which they are adopted as insulating, exterior, or partition constructions. Test bricks known as Earth Blocks Class 3 (EB3), which have a mean compressive strength value between 3.8 and 5 MPa, can be deployed as non-load-bearing, self-supporting walls. Bricks with a compressive strength value above 5 MPa, referred to as Earth Blocks Class 4 (EB4), can be used as inner walls and load-bearing walls of low-rise and mid-rise buildings [38].

Higher percentages of PPW content resulted in an increase in water absorption: 20.1% for 15% PPW compared to 13.9% for 0% PPW, which confirms that PPW increased the pore volume. Furthermore, the apparent porosity increased from 27.7% to 34.6% with an increase in the PPW content from 0% PPW to 15% PPW. A similar trend was observed with the incorporation of biosolids in fired clay bricks. The increase in the percentage of biosolids resulted in an increase in the rate of absorption due to the creation of pores during the firing process [39]. Water absorption values signify the long-term durability performance of the bricks. Therefore, excessively high values can lead to cracking because of the increase in the volume.

Among the tested percentages, the results show that the fired brick samples made with 10% PPW have optimal characteristics. Thus, the sample composed of 10% PPW was fired at three different temperatures (700 °C, 800 °C, and 900 °C). Table 6 summarizes the results of density, compressive strength, water absorption, and apparent porosity of bricks samples with 10% PPW fired at different temperatures. In general, increasing the PPW content in the mixtures decreased the specimen weights. Replacing clay (dense materials) with PPW (light materials) resulted in a total volume increase, even after compaction at 10 MPa. Increases in the compacted mix volume resulted in decreases in specimen weights and densities. Increasing the firing temperature of 10% PPW causes a linear increase in bulk density; such an impact on raw samples may result from the low bulk density level of PPW relative to that of clay and from the change in particle packing of the clay mix originated by incorporating a lightening additive, as well as the formation of a glassy phase. Compared to the sample with 0% PPW content, the addition of 10% PPW reduced the bulk density by 19.04% at 700 °C, 19.6% at 800 °C, and 18.7% at 900 °C.

D. Eliche-Quesada and Yuecheng reported similar findings, revealing that the incorporation of coffee grounds and cigarette butts in the development of masonry components decreases their compressive strength and load-bearing ability [40]. Therefore, increased loss of organic material during firing leads to a higher level of total porosity in clay. Pursuant to current regulations, the bulk density in bricks may not be lower than 1050 kg/m^3^. The bricks made with 10% PPW at 700 °C presented high porosity because the generation of gas from the decomposition of carbonaceous matter generates more porosity; in addition, the LOI of PPW bricks is higher than that of control bricks fired at 900 °C. This may be caused by the partial closure of open pores or by a decrease in the interconnectivity between pores due to the formation of a glassy phase at 900 °C.

Generally, the results show that as the percentage of PPW increased, the density of the bricks decreased, while the water absorption and apparent porosity increased. However, the compressive strength remained within acceptable limits, except for the bricks with 15% PPW. This study suggests that adding 10% PPW may be the best choice for achieving both the desired specifications and durability performance.

### 3.2. Thermal Conductivity Assessment of Bricks Incorporating Pomegranate Peel Waste

**Figure 6:**
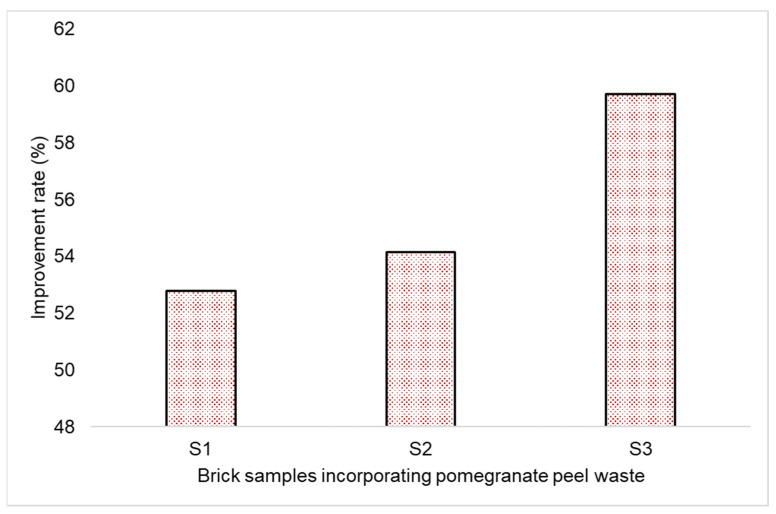
The rate of improvement in terms of thermal conductivity of the fabricated brick samples (S1–S3) compared to the conventional brick sample.

This study investigated the thermal conductivity values of bricks fabricated using pomegranate peel waste (PPW) as the primary ingredient and fired at different temperatures. The results show that the thermal conductivity values of the bricks decreased with increasing firing temperature. The decrease in thermal conductivity was attributed to the increased porosity of the bricks, which resulted from the development of larger pores in the brick structure when exposed to high temperatures. The highest thermal efficiency was observed in sample S3, which exhibited the lowest thermal conductivity value of 0.29 W/m.°C among all tested samples, as shown in Table 7. The rates of improvement in thermal conductivity values for S1, S2, and S3, when compared to conventional bricks, were 52.77%, 54.16%, and 59.72%, respectively, as shown in Figure 6.

The findings suggest that utilizing PPW bricks in building construction can be an effective approach to reducing energy consumption and minimizing carbon emissions. Additionally, the use of sustainable materials in building construction can contribute to the achievement of environmental sustainability and a reduction in the carbon footprint of the construction industry.

Moreover, the results of this study emphasize the importance of firing temperature in determining the thermal conductivity and porosity of the fabricated bricks. Higher firing temperatures were found to increase the porosity and decrease the thermal conductivity of the bricks, leading to improved thermal efficiency. However, excessive porosity can compromise the structural integrity of the bricks, indicating the need for careful control of the firing process to obtain the desired properties.

The findings of this study have significant implications for the development of sustainable building materials and design. The use of PPW bricks in building construction has the potential to contribute to the reduction in energy consumption and carbon emissions while also promoting the use of sustainable materials. This study also highlights the need for further research to explore the full potential of PPW bricks and to develop new and innovative building materials for sustainable building design.

### 3.3. Thermal and Energy Performance of the Investigated Building

#### 3.3.1. The Effect of the Investigated Bricks Made from Pomegranate Peel Waste on the Indoor Air Temperature

This study aimed to assess the insulation ability of bricks made from pomegranate peel waste against weather conditions. The tested brick wall samples were made from pomegranate peel waste and fired at different temperatures, then compared to a conventional brick case using Design Builder simulation software. The study was conducted on 21 June, the official longest day of the year in the northern hemisphere, as it is a day with high solar radiation and temperature fluctuations, as reported in previous studies [41]. The temperature of the air inside the building was monitored and recorded for conventional brick (BC) and the other brick wall samples under investigation. The results show that the installation circumstances of the pomegranate peel waste bricks significantly influenced the interior air temperature.

**Figure 7:**
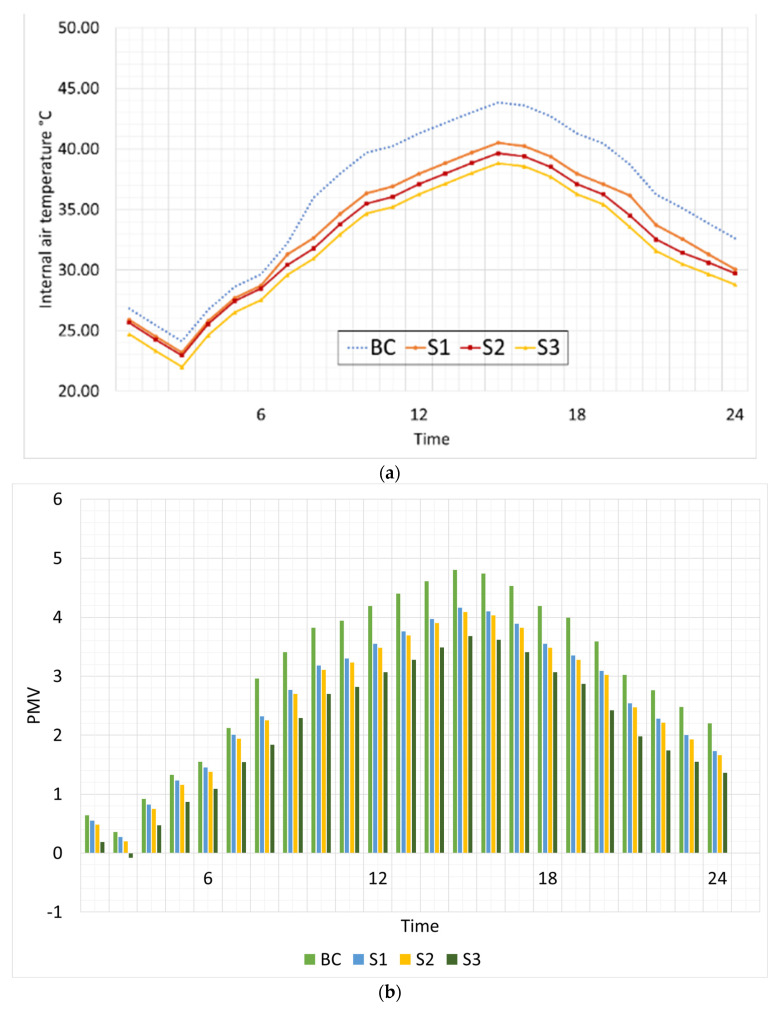
The simulated thermal conditions in the investigated building. (**a**) Indoor air temperature using varied brick samples; (**b**) thermal comfort using the PMV index.

Figure 7a displays the thermal efficiency of the proposed brick samples made from pomegranate peel waste and shows the possible reductions, along with the conventional brick used as a comparison. It was found that incorporating pomegranate peel waste into exterior brick walls considerably reduces interior air temperature fluctuations throughout the day, which has significant implications for energy efficiency and indoor comfort.

The thermal performance of the proposed brick samples made from pomegranate peel waste was found to be highly comparable, despite their evident variances. Among all the tested samples, S3 showed the largest reduction in interior air temperature, with a potential decrease of 2.07 to 5.02 K, making it significantly different from BC. However, S1 exhibited the least desirable thermal performance, with an average reduction in air temperature of only 0.93 K to 3.34 K, indicating that it had fewer benefits than any other brick sample. The thermal performances of S1 and S2 tended to be similar, which could be attributed to their comparable thermal conductivity. The greatest rate of improvement regarding internal air temperature was observed during daylight hours, with an average improvement ranging between 13% and 15% for S3, 10% to 13% for S2, and 7% to 9% for S1. These outcomes are in line with previous studies that have reported a significant enhancement in internal thermal performance through the integration of agricultural waste in fired bricks [6,9,13,14,19].

To assess the building’s thermal performance, Fanger’s predicted mean vote (PMV) was employed [41], which is a popular statistic used in studies of thermal environmental modeling, design, assessment, and management. PMV takes into account various parameters, such as air temperature, humidity, clothing insulation, and metabolic rate to evaluate the thermal comfort of occupants. PMV results were extracted from Design Builder simulation software according to ASHRAE and depending on the following equation:

PMV = [(0.303 ∗ e^−0.036 ∗ M^ + 0.028) ∗ (M − W) + (0.1 ∗ W)] − 3.05 ∗ 10^−3^ ∗ M ∗ (M − 58.15) − 0.42 (1)

where PMV is the predicted mean vote of thermal sensation, ranging from −3 (cold) to +3 (hot); M is the metabolic rate of the occupant, expressed in metabolic equivalents (met), with 1 met equaling the metabolic rate at rest (58.15 W/m^2^); W is the external work rate of the occupant, expressed in W/m^2^; and e is the vapor pressure of water vapor in the air, expressed in kPa.

The results show a wide variation in PMV prediction across all evaluated brick samples, with PMV following the same patterns as the indoor air temperature in the analyzed environments. Figure 7b displays a comparison of the tested brick samples for PMV. The PMV values of S3 were the lowest among all the tested brick samples, making it the most successful of the set. Specifically, at the morning and afternoon hours of 3:00, PMV levels in S3 were reported to be 0.1 and 3.72, respectively. S2 was the second-best choice after S3 for creating acceptable temperature conditions, with PMV levels between 0.2 and 4.09. According to these findings, the building’s interior environment met the standards set out by the Saudi rating system (MOSTADAM).

It is important to note that this study is subject to some limitations. First, the study was conducted in a specific climatic region and may not be representative of other regions with different weather conditions. Additionally, the study focused only on the optimum percentage of pomegranate peel waste (PPW) according to the mechanical and physical properties of the bricks and did not consider other factors, such as the long-term durability of the bricks. Therefore, further research is needed to fully evaluate the potential of using pomegranate waste in brick production under different climatic conditions and to assess the long-term durability of the bricks. Nonetheless, the study’s findings provide a valuable contribution to the field of sustainable building practices and offer a unique approach to reducing waste and promoting energy efficiency.

#### 3.3.2. An Analysis of Cooling Energy Demands

**Figure 8:**
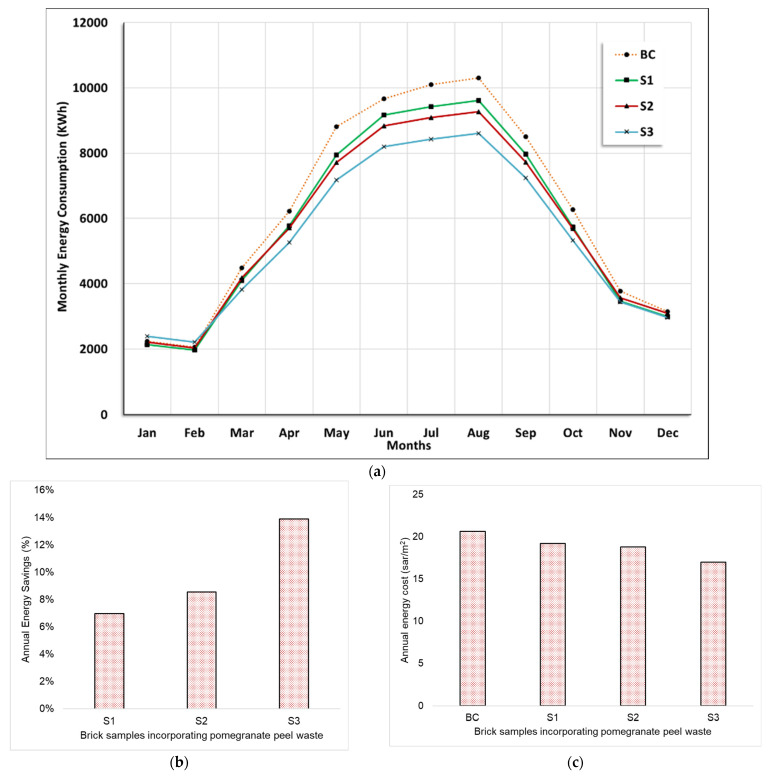
Simulation results for energy consumption attributed to the scenarios of polycarbonate windows: (**a**) monthly energy consumption; (**b**) annual savings; (**c**) annual costs.

This section presents an evaluation of the cooling energy requirements of buildings constructed with pomegranate peel waste (PPW) bricks using Design Builder modeling software. The simulation results illustrated in Figure 8 provide insights into how the firing temperature of PPW bricks affects the cooling energy requirements of the building. The results demonstrate that conventional bricks achieve poor cooling energy performance compared to PPW bricks, which can significantly reduce cooling energy usage.

It is noteworthy that the highest variation in energy use was observed during the summer months, between March and October, as shown in Figure 8a. During this period, cooling systems consume their annual maximum energy due to the high temperature in Jazan, which can reach up to 40 °C. Figure 8b shows that substituting conventional bricks with S3 can reduce the building’s cooling energy consumption by 10,499.65 kWh, corresponding to an improvement rate of approximately 13.89% compared to using conventional bricks as a base case. Bricks made from PPW fired at 800 °C reduced cooling energy consumption by 6457.05 kWh, representing an improvement of 8.54%. Similarly, firing PPW bricks at 700 °C reduced cooling energy usage by 5296 kWh, with an improvement rate of 6.97%. According to Figure 8c, the monthly cost of energy in SAR for a building located in Jazan City is lowest for the S3 brick sample and highest for the BC brick sample. The cost of energy decreases as the firing temperature of brick samples increases. Abdel Hamid et al. reported that using varying percentages of PPW ranging between 2.5% and 12.5% in building bricks can result in energy savings of approximately 23.3% [42]. In comparison, this study found that incorporating 10% of PPW material in building construction can lead to energy savings of about 13.89%. The results from both studies suggest that PPW has the potential to significantly reduce energy consumption in buildings in various climatic regions.

The findings of this study are significant because space cooling accounts for a substantial portion of a building’s overall energy demand, ranging from 37% to 42% [34,43]. Therefore, the use of PPW bricks in building construction could significantly reduce the energy usage of buildings, resulting in lower energy costs and reduced carbon emissions. The results of this study suggest that PPW bricks can be a viable alternative to conventional bricks in terms of reducing cooling energy consumption in buildings.

This study aimed to investigate the annual energy consumption and associated costs required for cooling in buildings constructed with different materials, including BC, S1, S2, and S3. The results demonstrate that the annual energy costs for cooling in the BC building were SAR 20.6/m^2^. In contrast, the buildings constructed with S1, S2, and S3 materials exhibited annual energy costs of SAR 19.2/m^2^, SAR 18.8/m^2^, and SAR 17/m^2^, respectively.

Comparison of the proposed building materials with the BC revealed that the use of S1, S2, and S3 materials resulted in significant reductions in energy consumption and associated costs for cooling. Specifically, the energy cost reductions were 7.02%, 8.97%, and 17.71% for S1, S2, and S3, respectively.

#### 3.3.3. Analysis of Pomegranate Peel Waste Bricks’ Potential Impact on Carbon Dioxide Emissions

The issue of increasing carbon dioxide (CO_2_) emissions in buildings over their entire life cycle is a growing concern. Despite technological advancements that have helped reduce CO_2_ emissions from building activities, embodied carbon dioxide in buildings has been on the rise in recent decades. CO_2_ emissions from utilities in buildings, such as heating, cooling, ventilation, and lighting, are a major contributor to this trend [44]. Embodied carbon dioxide [45] is influenced by various factors, such as resource extraction, manufacturing, transportation, construction, maintenance, and demolition. The materials and components used in buildings play a significant role in increasing the amount of CO_2_ in the atmosphere. Past research has linked CO_2_ emissions to air conditioning use in hot desert buildings. Studies have investigated various building materials, including window layouts, roofing tiles, and wall bricks, to improve thermal performance, reduce cooling energy use, and mitigate operational CO_2_ emissions. Such studies have highlighted the potential of using more sustainable building materials and design strategies to reduce the embodied carbon footprint of buildings. However, further research is needed to explore effective approaches for reducing embodied carbon dioxide in building construction and operation, especially in the context of changing climate conditions and evolving building codes and standards.

**Figure 9:**
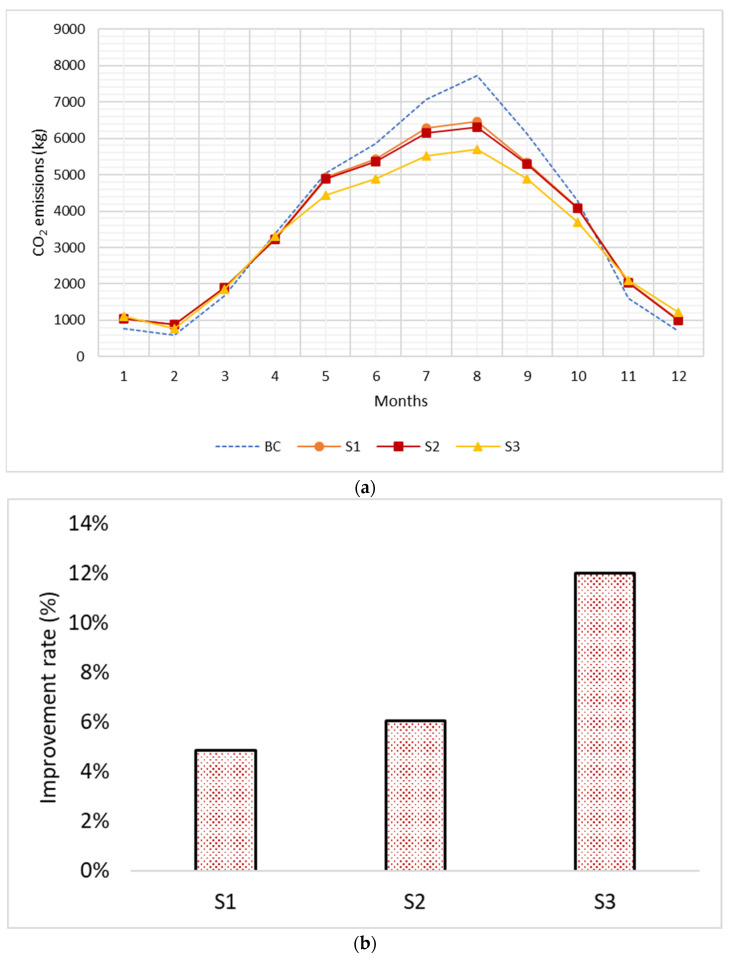
Simulation results: (**a**) monthly CO_2_ emissions; (**b**) improvement rate.

The results of this study show that CO_2_ emissions increase significantly during the summer season due to rising energy consumption for cooling purposes. August, the hottest month in Jazan City, had the highest CO_2_ emissions. This study evaluated the CO_2_ emissions associated with S1, S2, and S3 brick samples made from pomegranate peel waste (PPW) at firing temperatures of 700 °C, 800 °C, and 900 °C, respectively, using Design Builder modeling software. Among all tested samples, S3 exhibited the most efficient performance, with an improvement rate of 12% in reducing CO_2_ emissions. Samples S1 and S2 also showed a reduction in CO_2_ emissions, with improvement rates of 4.85% and 6.07%, respectively, as shown in Figure 9.

These findings suggest that using more energy-efficient materials for cooling purposes can significantly reduce CO_2_ emissions in buildings, particularly during the summer season. The use of PPW bricks fired at higher temperatures resulted in a lower embodied carbon footprint, which can be attributed to the reduction in cooling energy requirements. This study highlights the potential of using sustainable building materials, such as PPW bricks, to reduce the embodied carbon footprint of buildings.

However, it is important to note that this study only evaluated the CO_2_ emissions associated with cooling energy consumption and did not consider other factors that contribute to embodied carbon dioxide, such as transportation and maintenance. Further research is needed to comprehensively evaluate the embodied carbon footprint of buildings and explore effective strategies for reducing it.

In conclusion, the use of sustainable building materials such as PPW bricks has the potential to significantly reduce the embodied carbon footprint of buildings. The study findings demonstrate that the firing temperature of PPW bricks plays a crucial role in reducing cooling energy requirements and associated CO_2_ emissions during the summer season. This study highlights the importance of using more energy-efficient materials in building construction to mitigate the impact of buildings on the environment. The findings suggest that the use of PPW bricks in building construction can be an effective strategy for reducing the embodied carbon footprint of buildings, which is crucial in mitigating the impact of buildings on climate change. However, further research is required to fully evaluate the potential of using PPW bricks and other sustainable building materials in different situations and to explore effective approaches for reducing the embodied carbon footprint of buildings. This research is essential in promoting sustainable building practices and addressing the challenges presented by climate change and evolving building codes and standards.

### 3.4. The Advantages of Using Fabricated PPW Bricks: A Building Economic Study

The global trend towards energy conservation driven by high energy costs and environmental concerns has led to increased interest in sustainable building practices. While insulation of ceilings has been a common practice, exterior walls, despite their large surface area, have often been overlooked. This study aims to investigate the feasibility of using pomegranate waste as a substitute for clay in the production of conventional bricks, with the potential for significant cost savings. The objective is to explore the potential of this approach in contributing to energy conservation efforts and promoting sustainable building practices.

During the months of May to August, energy consumption exceeding 6000 kWh was observed in all studied samples, with the addition of two months (April and October) in BC. Consequently, all the aforementioned months were evaluated according to the second energy price category. Conversely, the remaining months were evaluated according to the first energy price category due to low energy consumption levels below 6000 kWh. The study involved the use of brick samples containing 10% pomegranate peel waste (PPW) with firing temperatures of 700 °C, 800 °C, and 900 °C for S1, S2, and S3, respectively. The impact of the firing temperature on production costs was deemed negligible, and therefore, the most efficient sample (S3) in terms of energy savings as compared to the conventional brick sample (BC) was investigated in terms of cost analysis.

The study findings indicate that S3, among all firing temperature options, has the potential to achieve energy savings of up to 13.89%. This promising result motivated us to explore the feasibility of producing bricks from pomegranate waste. While energy savings are an important factor, it is equally crucial to evaluate the financial benefits of such a production process. Therefore, a cost analysis was conducted, which involved determining the additional investment required for each suggested brick sample, as well as evaluation of the total annual energy cost savings in Saudi riyals (SAR).

This study assumed that the primary expenses for constructing both a conventional brick wall and the proposed PPW brick sample wall would be comparable, with a minor increase in the cost of the PPW brick sample due to the low cost of pomegranate peel waste. To determine the economic viability of the suggested PPW brick sample, we obtained production cost data (in SAR/m^2^) for both the conventional brick wall and the manufactured S3 brick sample from the local market, as shown in Table 8. The initial cost analysis considered the costs of materials and workers. In addition, the cost of transporting pomegranate peel waste (PPW) from Medina, which is considered one of the major cities in pomegranate cultivation and pomegranate juice production, to Jazan, the area under investigation, was determined using local market data, with a cost of SAR 1000 per 1000 m^2^ of PPW [46]. The labor costs per production rate (SAR/m^2^) were determined by dividing the total investment in the production process (SAR) by the production amount (m^2^). The data on labor costs were obtained from the statistics report provided by the Ministry of Human Resources and Social Development in KSA (HRSD) [47]. Based on the data presented in Table 9, we evaluated the annual energy costs, total construction costs, annual savings, and simple payback period (SPP) for each brick sample. The SPP was calculated as follows [48]:

$$\mathrm{SPP}=\frac{\mathrm{Additional}\;\mathrm{Investment}}{\mathrm{Annual}\;\mathrm{Saving}}$$

The study found that S3 demonstrated the potential to achieve substantial energy and financial savings, with a short SPP of 0.65 years. Notably, the additional investment required for the suggested brick sample was deemed minor, with a small increase in cost due to the low cost of pomegranate peel waste. The study’s findings indicate that S3 is a promising option for building owners seeking to reduce energy costs and enhance sustainability aspects. Finally, this study provides valuable insights into the potential of using pomegranate waste in brick production to achieve energy and financial savings while promoting sustainable building practices.

## 4. Conclusions

The purpose of this study was to investigate the potential effects of incorporating pomegranate peel waste (PPW) into brick clay at a concentration of 10% and firing at varying temperatures on the thermal and energy performance of the produced bricks. The study focused on evaluating the thermal parameters of density, thermal conductivity, and specific heat. Additionally, the mechanical and physical characteristics of the bricks were extensively tested. Based on the study findings, the following conclusions were drawn:

First, the addition of PPW to the brick samples resulted in enhanced thermal insulation properties by promoting the formation of pores, which directly reduced thermal conductivity.

Secondly, among all the fabricated brick samples, the insulation brick made from S3 and fired at 900 °C exhibited the most efficient thermal performance. It displayed a density of 1562 kg/m^3^, a specific heat of 1500 J/kg^−1^ K^−1^, and a thermal conductivity of 0.29 Wm^−1^ K^−1^.

Thirdly, S3 was found to be 13.89% more effective in preserving cooling energy, producing 12% less annual carbon dioxide emissions than conventional bricks. Additionally, it had the shortest simple payback period (SPP) of 0.65 years, indicating its cost-effectiveness. These findings are consistent with previous research [6,10,14] that has demonstrated a noteworthy enhancement in thermal properties, energy efficiency, and reduction in CO_2_ emissions, resulting in the shortest simple payback period (SPP).

This study provides recommendations for reducing thermal, energy, and total energy expenses and proposes the use of an environmental brick sample composed of 10% PPW fired at 900 °C instead of conventional bricks. Additionally, the study recommends that national authorities utilize the findings to improve national energy codes and insulation criteria for building envelopes. Finally, the study highlights the potential use of pomegranate peel waste in fired bricks for construction in hot and arid regions and its generalizability to other regions with similar climates. However, several limitations should be considered, such as resource availability and cost, construction suitability, and policy and circular economic factors. Further research is necessary to fully evaluate the feasibility and potential impact of using recycled PPW bricks. This should include an assessment of their environmental and economic implications; durability; thermal and energy performance; and potential for reuse, recycling, or repurposing. Regulatory frameworks, incentives, and market demand must also be considered.

## Figures and Tables

**Table 1:** Brick samples and their firing temperatures.

Sample	Firing Temperature °C
S1	700
S2	800
S3	900

**Table 2:** XRF of raw materials.

Oxide Composition	Pomegranate Peel Waste wt.%	Clay wt.%
CaO	10.48	0.50
SiO_2_	0.38	48.93
Al_2_O_3_	0.1	32.90
Fe_2_O_3_	0.91	1.19
SO_3_	0.4	0.29
Na_2_O	<0.01	0.09
P_2_O_3_	3.33	-
K_2_O	11.68	0.014
MgO	4.01	0.09
MnO	0.01	-
TiO_2_	0.1	5.92
CL	3.19	0.01
ZrO_2_	-	0.46
Cr_2_O_3_	-	0.14
LOI	65.35	9.2

**Table 3:** Study model input data.

Item	Specification
Building type	Residential building
Location	Jazan City—hot desert climate (Köppen: BSh)
Floor area (m^2^)	240
No. of floors	Ground floor and one floor
Floor height (m)	3.6
Occupancy (persons per building)	10
Window glazing	6 mm single clear glazing
Window-to-wall ratio	10%
Lighting (Lux)	400
HVAC	4 split air conditioning units for each flat
Cooling setpoint (°C)	25
Heating setpoint (°C)	18

**Table 4:** Residential electricity prices.

Bracket	Category (kWh)	Price (SAR)
First	0:6000	0.18
Second	more than 6000	0.30

**Table 5:** Average values for AP, CS, BD, and WA of bricks with different waste additives fired at 900 °C.

PPW (%)	AP (%)	CS (MPa)	BD (kg/m^3^)	WA (%)
0	27.7	18.5	1922.02	13.9
5	29.9	13.9	1713.45	14.7
7.5	32.9	11.2	1607.1	16.2
10	33.03	10.3	1562.9	17.5
15	34.6	4.6	1348.3	20.1

**Table 6:** Average values and standard deviations for AP, CS, BD, and CW of bricks with 10% PPW at different firing temperatures.

Firing Temperature (°C)	AP (%)	CS (MPa)	BD (kg/m^3^)	WA (%)
700	35.80	6.7	1480.8	18.6
800	34.04	7.97	1507.7	17.5
900	33.03	10.3	1562.9	17.3

**Table 7:** The characteristics of the investigated brick samples.

Sample	Firing Temperature (°C)	Thermal Conductivity W/m.°C	Specific Heat (J/kg^−1^.K^−1^)	Density (kg/m^3^)
Base case	900	0.72	800	1922
S1	700	0.34	2013	1480
S2	800	0.33	1800	1507
S3	900	0.29	1500	1562

**Table 8:** The material costs for both conventional and PPW brick samples.

Material	Material Unit Cost (SAR/m^2^)
Clay (kaolin)	6
Pomegranate peel waste	1.2
Cement mortar	4

**Table 9:** The calculated simple payback period associated with the brick samples.

	Wall Cost (SAR)	Additional Investment (SAR)	Energy Cost (SAR/Year)	Annual Savings (SAR/Year)	SPP (Year)
BC	22,628.39	0	20,795.34	0	
S3	25,454.97	2826.58	16,476.54	4318.8	0.65

## Data Availability

Not applicable.
